# Analysis of *Campylobacter jejuni* infection in the gnotobiotic piglet and genome-wide identification of bacterial factors required for infection

**DOI:** 10.1038/srep44283

**Published:** 2017-03-10

**Authors:** Stefan P. W. de Vries, Aileen Linn, Kareen Macleod, Amanda MacCallum, Simon P. Hardy, Gill Douce, Eleanor Watson, Mark P. Dagleish, Hal Thompson, Andy Stevenson, David Kennedy, Abiyad Baig, Chris Coward, Duncan J. Maskell, David G. E. Smith, Andrew J. Grant, Paul Everest

**Affiliations:** 1 Department of Veterinary Medicine, University of Cambridge, Madingley Road, Cambridge, United Kingdom; 2 University of Glasgow, Veterinary School, Glasgow, United Kingdom; 3 School of Pharmacy and Biomolecular Sciences, University of Brighton, United Kingdom; 4 Institute of Infection, Immunity and Inflammation, University of Glasgow, United Kingdom; 5 Moredun Research Institute, Edinburgh, United Kingdom; 6 Heriot-Watt University, School of Life Sciences, Edinburgh, Scotland, United Kingdom

## Abstract

To investigate how *Campylobacter jejuni* causes the clinical symptoms of diarrhoeal disease in humans, use of a relevant animal model is essential. Such a model should mimic the human disease closely in terms of host physiology, incubation period before onset of disease, clinical signs and a comparable outcome of disease. In this study, we used a gnotobiotic piglet model to study determinants of pathogenicity of *C. jejuni*. In this model, *C. jejuni* successfully established infection and piglets developed an increased temperature with watery diarrhoea, which was caused by a leaky epithelium and reduced bile re-absorption in the intestines. Further, we assessed the *C. jejuni* genes required for infection of the porcine gastrointestinal tract utilising a transposon (Tn) mutant library screen. A total of 123 genes of which Tn mutants showed attenuated piglet infection were identified. Our screen highlighted a crucial role for motility and chemotaxis, as well as central metabolism. In addition, Tn mutants of 14 genes displayed enhanced piglet infection. This study gives a unique insight into the mechanisms of *C. jejuni* disease in terms of host physiology and contributing bacterial factors.

*Campylobacter* spp. are recognised as the most common cause of acute food-borne bacterial diarrhoeal disease in humans, both in developed and developing countries^1^,^2^ with *Campylobacter jejuni* being responsible for ~85% of the human infections and *Campylobacter coli* accounting for ~15%. A spectrum of disease is seen in humans with clinical presentation ranging from watery to severe dysentery-like inflammatory diarrhoea^3^,^4^. In developed countries, diarrhoeal disease is manifested by a dysenteric-type illness that reflects the process of inflammation occurring within the gastrointestinal tract, findings that were reproduced in human volunteer studies using clinical isolates of *C. jejuni*^5^.

Our understanding of the bacterial components/mechanisms responsible for infection of the human gastrointestinal tract is far from complete. A significant obstacle to elucidating the pathophysiology of *Campylobacter* infection is the lack of a robust animal model that accurately reproduces the characteristics of gastroenteritis seen in human infection^6^,^7^,^8^.

*C. jejuni* and *C. coli* are able to colonise specific pathogen free pigs, however, they are limited in their ability to cause clinical signs of disease^9^. However, earlier work in the 1980s and 1990s demonstrated the potential of the gnotobiotic piglet model of *C. jejuni* infection to study the pathogenesis of diarrhoeal disease^10^,^11^,^12^. Gnotobiotic piglets are derived *via* hysterectomy and are deprived of colostrum, and consequently they lack maternal antibodies and a competing microflora. In the model reported by Vitovec *et al*.^10^, one-day old gnotobiotic piglets were orally infected with *C. jejuni*. Clinical manifestation of enteric disease was observed at day 4–5 post-infection, with signs of disease confined to the large intestine, these included inflammation, oedema and influx of neutrophils^10^. Transmission electron microscopy showed disruption of the intestinal lining with dilated intercellular space between enterocytes^10^. In a study by Boosinger and Powe, *C. jejuni* was also detected on the surface of epithelial cells as well as sub-mucosal^12^. These studies demonstrated that gnotobiotic piglets represent a suitable model to investigate disease mechanisms of *C. jejuni* as their gastrointestinal anatomy and physiology is similar to humans as well as their susceptibility to many enteric pathogens^6^,^7^,^8^,^10^,^12^,^13^,^14^.

The availability of various genome-wide screening methods employing next-generation sequencing technologies enabled detailed assessment of the molecular mechanisms involved in colonisation of chickens and the invasion of epithelial cells^15^,^16^. However, no such studies have been performed in models mimicking human campylobacteriosis.

In this study we have re-established and characterised in detail a gnotobiotic piglet model to investigate diarrhoeal disease caused by *C. jejuni* and combined transposon (Tn) mutagenesis with Tn-seq (transposon insertion site sequencing) to identify genes implicated in infection of the piglet gastrointestinal tract.

## Results and Discussion

### Characterisation of the gnotobiotic piglet model of *C. jejuni* infection

**Figure 1:**
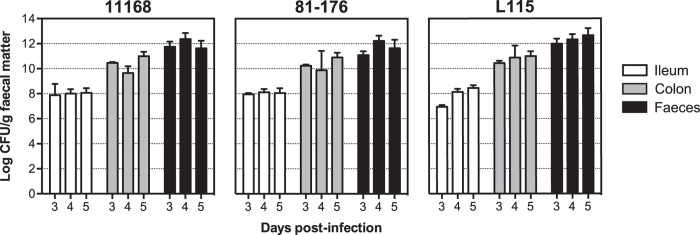
High bacterial loads in faeces of gnotobiotic piglets infected with *C. jejuni*. Bacterial load (Log CFU/g) in faecal matter from the ileum, colon and faeces of piglets at days 3, 4 and 5 p.i. with *C. jejuni* strains 11168, 81–176 and L115. Data shown are means with SD.

Piglets were surgically delivered through hysterectomy *via* a sterile plastic bubble into a sterile isolator and fed a sterile diet, consequently the piglets are gnotobiotic and thus lack a commensal flora. One day post-delivery, piglets were orally challenged with ~5 × 10^9^ colony forming units (CFU) of *C. jejuni* strains 11168, a human enteritis isolate^17^, 81–176, a human campylobacteriosis outbreak isolate^18^, or strain L115 that was isolated from a paediatric diarrhoeal disease case^19^. All three tested *C. jejuni* strains did successfully establish infection of the gnotobiotic piglet gastrointestinal tract at day 3 post-infection (p.i.), with bacterial loads at 5 days p.i. reaching approximately 10^8^ CFU/g of ileal content, 10^10^ CFU/g of colonic content, and 10^12^ CFU/g in the faeces collected from the last inch of the rectum (Fig. 1). Bacterial loads were found to be higher in the colon and faeces than in the ileum.

**Figure 2:**
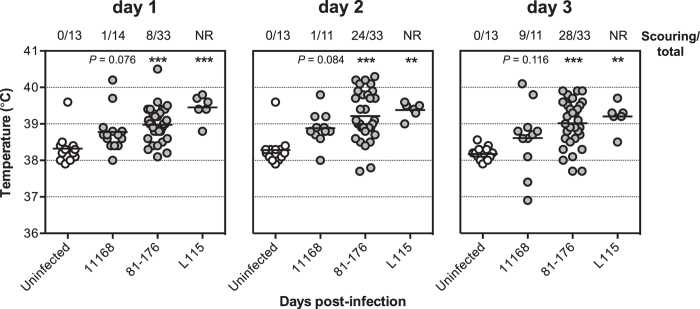
Increased rectal temperatures after infection of piglets by *C. jejuni*. Piglets were infected with *C. jejuni* and rectal temperatures were measured on days 1, 2 and 3 p.i. At the top of the graphs, the number of animals with scours is indicated, over the total number of piglets per group. Statistical significance was analysed using an Kruskal-Wallis test and Dunn’s multiple testing correction with **P < 0.01 and ***P < 0.001. NR = not recorded.

Despite comparable bacterial loads for all three *C. jejuni* strains, the piglets infected with strains 81–176 and L115 developed increased rectal temperatures at day 1, 2 and 3 p.i. whereas this effect was less pronounced after infection with 11168 (Fig. 2). At day 3 p.i., 9 out of 11 piglets infected with 11168 suffered from watery diarrhoea (scour) and 28 out of 33 piglets infected with 81–176 (Fig. S1). Over the course of the experiment (5 days), infected piglets failed to put on weight (failure to thrive) or lost weight compared to uninfected gnotobiotic animals. These clinical characteristics resemble those observed in *C. jejuni* enteritis in experimental studies in humans and in natural human cases^5^.

**Figure 3:**
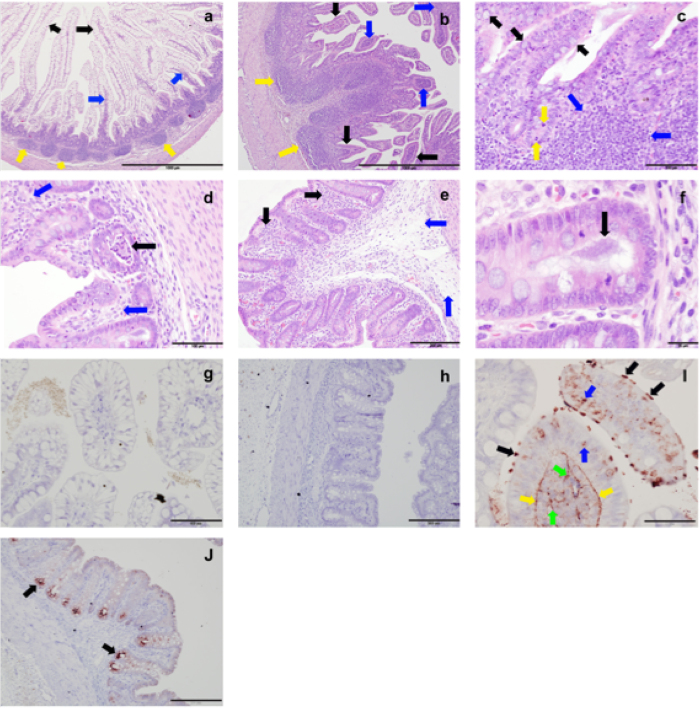
Histology of the piglet intestine infected with *C. jejuni*. Tissues were obtained on day 4 (**a**–**f**) or 5 (**i**,**j**) p.i. with *C. jejuni* 81–176 (**b**–**f**,**i**,**j**) or from uninfected gnotobiotic control piglets (**a**,**g**,**h**) and analysed using haematoxylin-eosin staining (**a**–**f**) or immuno-labelling of (red chromogen) of *C. jejuni* with a haematoxylin counterstain (**g**–**j**). (**a**) Uninfected ileum; long villi extend deep into the lumen, which is populated by cells with vacuolation of cytoplasmic contents (black arrows), the low cellularity of the lamina propria (blue arrows) and the presence of discrete Payer’s patches (yellow arrows). (**b**) Infected ileum; villi are shorter and blunter (black arrows) than in uninfected controls, the lamina propria is highly cellular (blue arrow) due to lymphocyte infiltration but also smaller numbers of eosinophils and occasional neutrophils and the sub-mucosal lymphoid tissue (Peyer’s patches) are greatly expanded and coalescing (yellow arrow). (**c**) Higher magnification of panel b. Larger numbers of mucous cells within the epithelium of the villi (black arrows), large numbers of lymphocytes in the sub-mucosa (blue arrow) and cells undergoing mitosis within the epithelium in the depths of the crypts (yellow arrows). (**d**) Infected caecum; crypt abscesses (black arrow) and oedematous lamina propria (blue arrow). (**e**) Infected colon; oedema within the lamina propria and sub-mucosal tissue (blue arrows) and a mild lymphocyte infiltration (black arrows). (**f**) Infected colon; bacteria in the lumen of the base of an intestinal crypt (black arrow). (**g**) Uninfected ileum; some non-specific staining of ileal contents, however no bacterial staining was detected and the pattern of staining was different from the infected animals. (**h**) Uninfected colon. (**i**) Infected ileum; *C. jejuni* microcolonies on epithelial cell microvilli (black arrows), *C. jejuni* in the cytoplasm of epithelial cells (blue arrows), along the basement membrane of the epithelium (yellow arrows) and within the lamina propria (green arrows). (**j**) Infected colon; *C. jejuni* was primarily found in crypt epithelial cells (black arrows). Scale bars represent 1,000 μm (**a**,**b**), 100 μm (**c**,**d**,**g**), 200 μm (**e**,**h**,**j**), 20 μm (**f**) and 50 μm (**i**).

There was little evidence of severe inflammatory enterocolitis in the histological assessment of piglets that developed diarrhoea (Fig. 3). Compared to uninfected controls (Fig. 3a), histology on ileal tissue from infected piglets showed relatively shorter and blunter villi with mitotic figures in the crypts, an increase in mucous cells, and a minimal neutrophil infiltrate into the lamina propria and expansion of sub-mucosal lymphoid tissue as denoted by well populated Peyer’s patches (Fig. 3b,c). There was an increase in mucous secretory cells, evidence of shortening and blunting of villi and some villous cells showing damaged nuclei (Fig. 3b,c). The vacuolation of mucosal epithelial cells, especially those at the apex of villi (Fig. 3b,c), was most likely due to the high fat content of the diet. In the caecum some crypt abscesses were present (Fig. 3d). In the colon of the infected piglets there was oedema in the lamina propria and some tissue damage compared to uninfected controls (Fig. 3e,f), in line with findings reported by Vitovec *et al*.^10^. Typical colonic findings included occasional bacterial colonies present, pyknotic nuclei and a mild increase in cellularity in the lamina propria, due to predominantly lymphocytic infiltration with the occasional polymorphonuclear leukocytes (PMN) also present (Fig. 3b,c). Detailed examination of the ileal tissue using immunohistochemistry specific for *Campylobacter* spp. showed the presence of *C. jejuni* microcolonies on the villi and revealed the presence of intracellular bacteria in the villous lamina propria (Fig. 3g,h).

**Figure 4:**
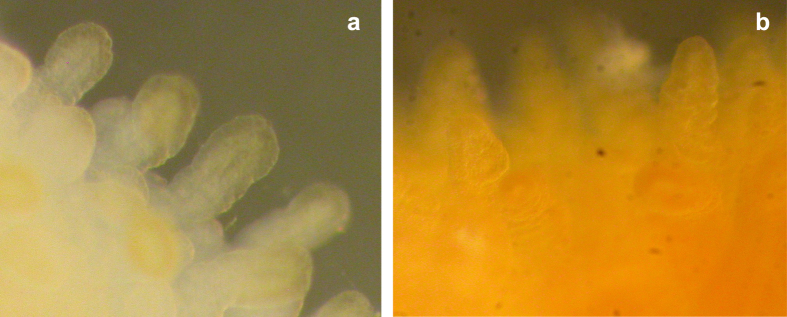
*C. jejuni* infection affects villi structure and bile staining in ileal tissue. Light microscopy examination of ileal tissue taken immediately after post mortem 4 days p.i. with *C. jejuni* strain 81–176. (**a**) Rounded blunt villi and reduced bile staining was observed in ileal tissue from infect piglets. (**b**) In uninfected piglets the villi were pointed and a clear bile staining was observed.

Features associated with *C. jejuni* 81–176 infection also include the gross changes in colour of the infected tissue and the shape of the villi compared to uninfected tissue (Fig. 4). Infected tissues were noticeably paler than control tissues, showing reduced bile-staining together with shortened and broad villi, whereas uninfected piglet ileum exhibited normal, ‘leaf-like’ villous architecture and brown, bile-stained mucosa. This suggests that *C. jejuni* infection prevents bile re-absorption in the ileum. In mammals, resorption of 90% of the bile acids released from the gall bladder after a meal occurs in the ileum. When not re-absorbed excess bile acids cause acute watery diarrhoea^20^. Bile acid malabsorption (BAM) is a well-known syndrome that usually results in chronic watery diarrhoea, with excess faecal bile acids. Furthermore, bile constituents have been shown to induce pathogenicity determinants of *C. jejuni* consistent with a role for decreased bile re-absorption enhancing colonisation^21^.

**Figure 5:**
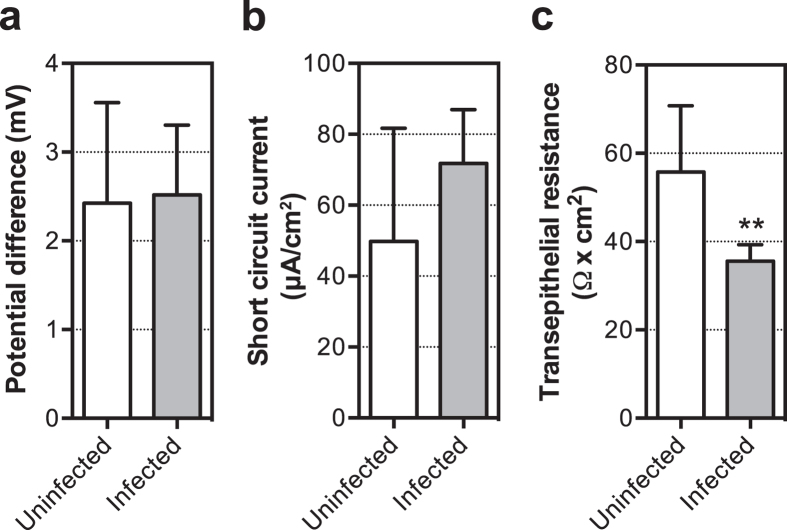
Reduced tissue resistance after *C. jejuni* infection in gnotobiotic piglets. Analysis was performed on tissue derived from piglets infected with *C. jejuni* strain 81–176 (4 days p.i.) and uninfected control animals in an Ussing Chamber model. No difference in potential difference (**a**) or change in short circuit current (**b**) across mucosa was observed between infected or uninfected samples, however tissue resistance (**c**) was significantly reduced in infected animals, indicating a more leaky bowel. Statistical significance was analysed using a two-tailed Mann-Whitney (non-parametric) test with **P < 0.01.

The observed changes to the morphology of villi in infected piglets may reflect an alteration of the barrier function of the small bowel. In order to assess this, we conducted a series of Ussing chamber experiments to determine changes in short circuit current and transepithelial resistance across muscle stripped ileum as a measure of the secretory component and paracellular leak, respectively. No changes were observed in potential difference (Fig. 5a), or short circuit current (Fig. 5b), indicating that there was no active secretory component to the *C. jejuni* induced diarrhoeal disease. *i.e*. net secretion of chloride ions across the apical membrane of the ileum. Piglets infected with *C. jejuni* strain 81–176 showed loss of tissue resistance over the course of acute diarrhoeal disease compared to uninfected controls (Fig. 5c), indicating that the tight junctions of the ileum were leaky. Thus *C. jejuni* appears to inhibit absorption of water from the ileum in infected animals rather than to up-regulate secretion.

### Genome-wide detection of genes implicated in piglet infection

We used the gnotobiotic piglet model to conduct a genome-wide Tn mutant library screen to identify genetic factors important for infection. Previous Tn mutant screens in chicken colonisation models showed extensive random dropout of colonisation-proficient Tn mutants and extensive between-experiment variation^15^,^22^,^23^,^24^, for example Johnson *et al*., reported a 51% loss of the Tn mutants present in the input pool after recovery from colonised 1-day-old chickens^15^. Unpredictable variations in population structure were even observed when birds were inoculated with just two competing wild-type isogenic-tagged strains (WITS) that have indistinguishable phenotypes in pure culture^25^. To determine whether this also applies to the gnotobiotic piglet model, piglets were infected with two *C. jejuni* M1 WITS that can be distinguished by a DNA tag inserted into a pseudogene. At various time-points the abundance of each WITS was determined by quantitative PCR (qPCR) and a log_10_ competitive index (CI) was calculated; a log_10_ CI score of 0 indicates that any confounding effects are unlikely to be present. Both after oral and rectal infection with two *C. jejuni* M1 WITS given as a single inoculum in a 1:1 ratio, both WITS were reliably recovered from piglets (Fig. S2). During the course of 4 days of infection, any bias towards one WITS was less than half a log (Fig. S2). These findings contrast sharply with previous chicken colonisation studies where biases of up to four orders of magnitude were seen when WITS were inoculated at 1:1 ratio^25^, and indicates that the gnotobiotic piglet model is suitable for screening a Tn mutant library.

To identify genes implicated in infection of piglets, the developed model was used to screen a *mariner* Tn mutant library in *C. jejuni* strain M1cam^22^,^26^. Consisting of 9,951 unique Tn insertion mutants. Two groups of piglets (isolator 1: *n* = 11 and isolator 2: *n* = 10) were infected with 10^9^ CFUs of the Tn mutant library. Tn mutants that populated the gastrointestinal tract were recovered at five days p.i. To identify genes required for infection, the composition of the Tn mutant library in the inoculum (input) and the library recovered from infected piglets (output) was determined by Tn insertion site sequencing, referred to as Tn-seq^27^. Tn-seq read-out detected a total of 8,761 (7,414 ± 477) unique Tn mutants covered by more than 10 sequence reads in the input replicates (*n* = 3), with 999 out of 1,654 genes in the *C. jejuni* M1cam genome being represented by 1 or more Tn insertion mutants. For reference, a total of 512 genes in the *C. jejuni* M1cam genome are indispensable for *in vitro* growth (for more details see Methods and ref. 22), hence these were eliminated from downstream analyses. A total of 8,340 (5,484 ± 3,332) unique Tn mutants were detected in the output pools recovered from the piglets (isolator 1 and 2), representing 96% recovery of the Tn mutant library complexity from infected piglets. More specifically, 7,320 (5,136 ± 2,012) and 7,621 (5,395 ± 969) unique Tn mutants were recovered from piglets housed in isolator 1 and 2, respectively. On average, recovery of 74% of the Tn mutants were recovered per infected piglet. This is likely the consequence of mutants being lost due to their inability to infect the piglet gastrointestinal tract and/or loss due to an infection bottleneck. Our results indicated a reliable recovery of a large part of the Tn mutant library from infected gnotobiotic piglets and suggest that there may be no, or only a minor, infection bottleneck.

**Figure 6:**
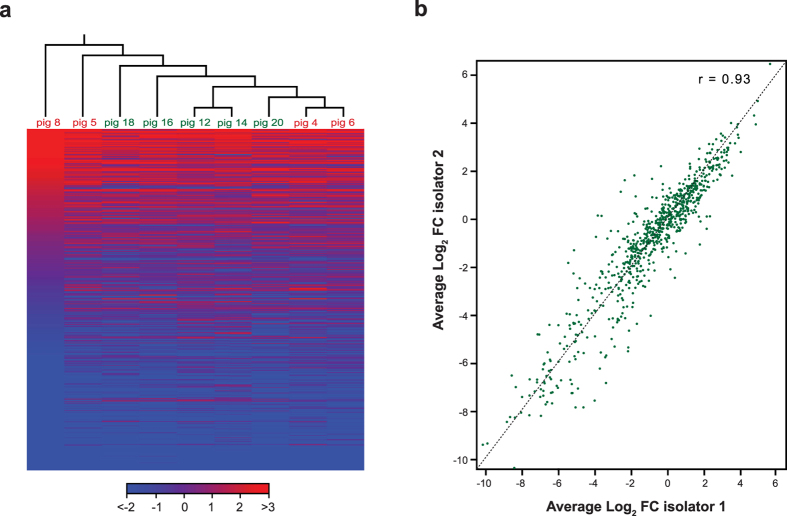
Tn mutant library behaviour in piglets housed in two separate isolators. (**a**) Hierarchical clustering of log2 fold-change per gene (output/input) demonstrated the absence of isolator-specific clustering. (**b**) Scatter plot of log2 fold-change per gene (input/output) for isolator 1 versus isolator 2 revealed a strong correlation between the two isolators. Piglet 8 was found to be responsible for the largest variance within the sample set, this was due to a lower number of Tn mutants being recovered from this piglet compared to others.

First, we analysed whether there was a difference in the behaviour of Tn mutants within isolator 1 and 2. A principal component analysis (PCA) based on the read counts per individual Tn mutant showed clustering of the input samples and indicated that there was no isolator-specific clustering of piglets (Fig. S3a). Both PCA and hierarchical clustering based on log_2_ fold-change per gene (output/input; average of inputs *n* = 3) also indicated the lack of isolator-specific clustering (Fig. S3b and Fig. 6a). We observed that piglet 8 showed the largest variability within the data set (Fig. S3a,b and Fig. 6a), this was likely to be the result of fewer Tn mutants (3,988) being recovered from this piglet compared to the other piglets. Importantly, there was a strong correlation (Pearson correlation of 0.93) between the log_2_ fold-change per gene for isolator 1 and 2 (Fig. 6b).

Next, genes required during infection were identified using data combined for piglets from both isolators. For this, we excluded genes that when inactivated by a Tn insertion displayed attenuated growth, referred to as “fitness” genes (for more details see Methods). Of the 1,140 non-fitness genes, only the 763 genes that were covered by more than 100 reads (combined for all Tn insertions in the gene) in the input (average read count, *n* = 3) were considered for further analysis. The Tn-seq screen identified 123 genes required for piglet infection as their corresponding Tn mutants were negatively selected from the Tn mutant library population, defined as a log_2_ fold-change of <−2 between output (recovered from pigs) and input (Benjamini and Hochberg adjusted *P* < 0.05) and a minimum of two Tn mutants per gene that showed a log_2_ fold-change of <−2; log_2_ fold-change cut-offs were determined based on a MA-plot (Fig. S4). A total of 14 genes were identified of which Tn mutants showed enhanced survival, with a log_2_ fold-change of >3-fold and >2 Tn mutants with a log_2_ fold-change of >3. It must be noted that genes that encode secreted proteins such as the Campylobacter invasion antigen B (CiaB)^21^ can be complemented *in trans* and hence are not identified in Tn library screens as shown here and by Gao *et al*., during the invasion of human gut epithelial tissue culture cells^16^. A detailed overview of the Tn-seq analysis and genes of which mutants were negatively or positively selected during piglet infection is presented as Table S1.

**Figure 7:**
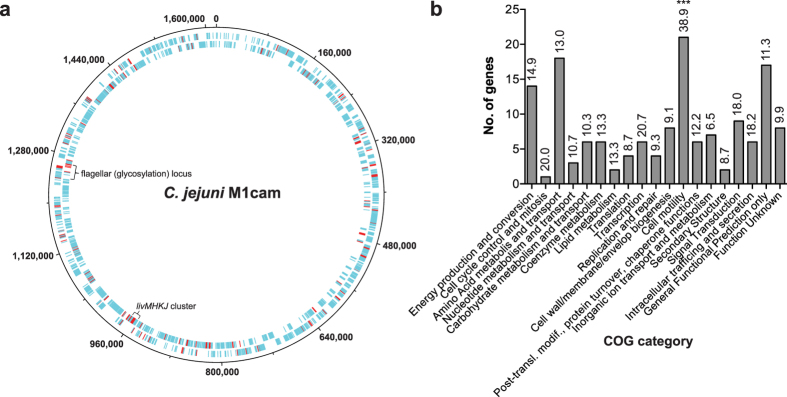
Candidate genes implicated in growth in the pig gastrointestinal tract. (**a**) Circular diagram of the *C. jejuni* M1cam genome with genes required during pig infection indicated in red. No distinct clustering was observed except for several genes located in the flagellar (glycosylation) locus and genes encoding an ABC-type branched-chain amino acid transporter system (*livMHKJ*). (**b**) Functional class enrichment analysis of genes linked to piglet infection. Bars display the number of genes assigned to each COG category that were required during infections of pigs, with the percentage of genes per COG given on the top of each bar. COG functional class enrichment was analysed using a Fisher-exact test and corrected for multiple testing using Q-value^50^, ***Q < 0.001.

Among the genes of which Tn mutants were negatively selected during infection of piglets were 26 flagellar system genes, which included genes involved in flagellar glycosylation, *pseA, pseB, pseD (maf3*), *pseE*, and *maf*-*5*^28^,^29^,^30^ (Fig. 7a). Limited chromosomal clustering of genes implicated in piglet infection was observed, except for genes in the flagellar (glycosylation) system (Fig. 7a). Functional enrichment analysis based on Cluster of Orthologous Groups (COG) categories highlighted a central role for motility, *i.e*. the COG category “cell motility” was the only category that was enriched amongst genes required during piglet infection (Fig. 7b). Further, several chemotaxis genes (*mcp4*_2, *mcp4_3, mcp4_4* and *cheR*) were identified, as well as genes implicated in *N*-linked glycosylation of membrane proteins (*pglF* and *pglH_capM*)^31^ and a putative capsular polysaccharide heptosyltransferase gene (CJM1cam_1374).

Central to the physiology of *C. jejuni* is its ability to utilise amino acids and organic acids such as lactate, pyruvate, acetate and tricarboxylic acid (TCA) cycle intermediates, reviewed in refs 32 and 33. We found that genes involved in these metabolic pathways may contribute to infection of the piglet gastrointestinal tract, although caution must be taken, as the availability of substrates/metabolites is dependent on the diet the piglets were fed on, *i.e*. the gnotobiotic piglets were fed a high-glucose evaporated milk diet (Carnation milk), and the metabolic conversions conducted by the resident microorganisms. This may be different in the gnotobiotic piglet compared to conventional piglets that posess a commensal microbiota. Amongst others we identified genes involved in lactate utilization (*lutA*_1; lactate to pyruvate), the lower part of the Embden-Meyerhof-Parnas pathway (*pyk*; pyruvate kinase and *pycA*; acetyl-CoA carboxylase), and in conversion of TCA cycle intermediates such as citrate (*gltA*), succinyl-coA (*sucC*), succinate/fumurate (*frdA* and *frdC*) and malate (*mdh*). The oxidation of succinate to fumurate is a key step in the oxidative TCA cycle and is primarily driven by fumurate dehydrogenase^34^. Interestingly, the importance of fumurate dehydrogenase was previously demonstrated using chicken colonization, with fumurate dehydrogenase genes (*frdAB*) being up-regulated during chicken colonization^35^ and *frdA* gene deletion mutants in *C. jejuni* 11168 show reduced chicken colonization^34^.

Linked to the TCA cycle are genes responsible for secretion or uptake of acetate (*ackA*; acetate kinase and *pta*; phosphate acetyltransferase); the direction of this pathway (anabolic or catabolic) is likely dependent on the growth-phase and nutrient availability^36^. Of these substrates, pyruvate, fumarate, succinate, malate and lactose have been reported to be chemoattractants for *C. jejuni*^37^. Of note, we observed that some genes, *i.e*. genes that encode PEP carboxykinase (*pckA*) and aconitase (*acnB*) in these central metabolic pathways were not identified in our screen due to them being required for full “fitness” (See Methods for further details).

Serine, aspartate, glutamate and proline are primary nutrient sources for growth of *C. jejuni*, which can be acquired during growth in the gastrointestinal tract. These amino acids are also in abundance in the evaporated milk-based diet^38^ that the piglets were fed on. Aspartate and serine are considered the preferred amino acids for growth, followed by glutamate and proline^32^,^33^, of which asparate, serine and glutamate are chemoattractants. In our screen we identified genes implicated in uptake of aspartate and glutamate (*peb1a* and *artM_*2/*pebC*) and TCA cycle influx of aspartate (*aspA*; conversion of aspartate to fumarate). In *C. jejuni* 11168, *aspA* was up-regulated during chicken colonization^35^, and an *aspA* gene deletion mutant in *C. jejuni* 81–176 was previously shown to be unable to use any amino acids except serine and was defective in colonization of chickens^39^. Interestingly, Tn inactivation of the serine utilization genes *sdaA* and *sdaC* (serine to pyruvate) appear to enhance piglet infection, this is in contrast to severely attenuated colonization of gastrointestinal and systemic tissues in a murine colonization model by *C. jejuni* strain 81–176 *sdaA* and *sdaC* defined gene deletion mutants^19^. This might be due to different substrate availability in piglets or related to strain-dependent metabolic preferences^40^. Branched-chain amino acids (BCAA) are linked to chemotaxis, *i.e*. isoleucine interacts with the transducer-like protein 3 (Tlp3)^41^; the LIV (leucine, isoleucine, and valine) ABC-type BCAA transport system (*livM, livH, livK*, and *livJ*), isoleucine and valine biosynthesis (*ilvH* and *ilvC*), and homoserine dehydrogenase (*hom*; reduction of aspartate to homoserine, which is an intermediate in isoleucine biosynthesis), were implicated in piglet infection. *C. jejuni* harbors various pathways for anaerobic respiration, of which the genes that encode formate (*fdhD* and *fdhG*) and nitrate (*napD*) dehydrogenases were required during infection. Further, the genes of which products handle oxidative stress (*catA*; catalase) and acid stress (*clpB*; Clp protease ATP-binding subunit) appear to be important for the ability of *C. jejuni* to establish piglet infection^42^.

## Conclusions

In this study, we set out to establish and characterize a gnotobiotic piglet model of gastrointestinal infection by the food-borne pathogen *C. jejuni*, with the aim to expand our knowledge about the molecular mechanisms underlying the lifestyle of *C. jejuni* during infection of host organisms.

In the gnotobiotic piglet model, infection with *C. jejuni* caused increased rectal temperatures and acute watery diarrhea with minimal inflammation. *C. jejuni* adhered to the luminal surface of the piglet small intestinal mucosa as microcolonies and localised intracellularly in the lamina propria and ileal crypts. In the piglet colon, the bacteria localised to the crypts, with only a few bacteria being present on the colonic luminal surface. Muscle-stripped ileum from the infected piglets had reduced transepithelial resistance compared with uninfected control tissue. This indicates that the tight junctions controlling paracellular fluxes of ions and water has been compromised since there was no increase in short-circuit current under a chloride gradient (representing transcellular ion movement of ions). This is consistent with similar results obtained in Caco-2 cells infected with *C. jejuni*^43^ and suggests that the lower small bowel in the infected animal will have a reduced absorptive ability than normal^44^. In addition, we found a greatly reduced or complete lack of bile staining in *C. jejuni*-infected animals. This indicates that *C. jejuni* infection may prevent bile acid re-absorption and that the excess of bile acids in the distal small intestine of infected piglets contributes to acute watery diarrhoea.

To understand the lifestyle of *C. jejuni* during infection of the piglet gastrointestinal tract at a molecular level, a genome-wide Tn mutant library screen was performed. The Tn library screen highlighted a crucial role for flagellar-mediated motility, chemotaxis and central metabolic pathways required to catabolise organic acids such as lactate, pyruvate, acetate, as well as TCA cycle intermediates and amino acids, mainly aspartate. Thus, it appears that the molecular mechanisms involved in *C. jejuni* intestinal infection of gnotobiotic piglets, overlap largely with those implicated in colonization of natural reservoir host organisms such as chickens but also with those required for invasion of human gut epithelial tissue culture cells. Our recent work revealed that 65 out of 172 genes required for colonisation of commercial broiler chickens and 34 out of 57 genes required for invasion of Caco-2 cells and were also found to play a role during the infection of gnotobiotic piglets^22^. The large overlap suggests that this model presents an opportunity to inform us about the mechanisms of disease in humans, however a detailed molecular and immunological characterisation of the model system would be required, for example through monitoring the transcriptional response of *C. jejuni* and the host during infection. Future work on elucidating the link between nutrient availability, chemotaxis, motility and colonisation/infection of host organisms will contribute to our understanding of *C. jejuni* pathogenesis.

In summary, these results underline the potential of the gnotobiotic pig model of *C. jejuni* intestinal infection to study molecular mechanisms of infection and host-pathogen interaction in a model that is clinically relevant and mimics human disease.

## Methods

### Bacterial strains, media and growth conditions

*C. jejuni* strains were cultured in Mueller-Hinton (MH) broth or MH blood agar (Oxoid) and incubated at 37 °C in a variable atmosphere incubator (VAIN, Don Whitley Scientific) in an atmosphere of hydrogen 6%, carbon dioxide 5%, oxygen 5% and nitrogen 84%. *C. jejuni* M1cam was cultured on Brain Heart Infusion (BHI, Oxoid) agar plates supplemented with 5% defibrinated horse blood (Oxoid) and 5 μg/ml trimethoprim (TrM). The *mariner* Tn mutant library was cultured in the presence of 10 μg/ml chloramphenicol (Cm). Bacterial strains and plasmids used in this study are detailed in Table S2.

### Piglet infection model

Gnotobiotic piglets were surgically delivered by hysterectomy through a sterile plastic bubble into a sterile isolator (Bell Isolation Systems Ltd, Livingston, UK). The piglets navels were treated with a strong iodine solution and the navels tied with cotton tape to stop blood loss. Piglets were ear tagged for identification 3 h post-delivery (p.d.). All piglets had an iron injection (deep intramuscular injection on the rump) at 24 h p.d., because of the sterile diet they are fed on, which is low in iron. The piglets were bottle fed for the first 24 hp.d. using a 1:1 mixture of Carnation milk (Nestle, Switzerland) and 10% autoclaved glucose solution. After 24 hp.d. the piglets were bottle fed a 1:1 mixture of Carnation milk and autoclaved water. At 3 days p.d. the piglets were introduced to trough feeding within the isolators. The piglets were subsequently fed on sterile Carnation milk plus B vitamin supplements within the isolators, and had access to sterile water at all times for the duration of the study.

Piglets were orally infected one day p.d. with 5 × 10^9^ CFU of *C. jejuni* strains 11168, 81–176 and L115; the dose was chosen based on the published literature of similar studies^10^,^12^,^14^. Rectal swabs were taken daily before and after the onset of diarrhoea to detect *C. jejuni* in faeces. *C. jejuni* WITS were inoculated by both oral and rectal gavage. For the *C. jejuni* M1cam Tn mutant library screen 4.7 × 10^8^ cfu/ml was administered by oral gavage per piglet in a 1 ml volume. Piglets were examined at relevant times post infection (p.i.) for clinical signs including increased rectal temperature, diarrhoea, decreased movement and hunching. They were clinically monitored daily with rectal temperatures taken and the extent of scouring recorded. On day 5 p.i. animals were euthanized by lethal injection of Pentobarbitone sodium B.P. (approx. 200 mg/kg, Rhone Merieux) and both ileal and colonic tissue were sampled for microbiological, physiological and histopathological analysis.

Approximately 1 gram of weighed faeces per sample was homogenised and diluted for a surface plate viable count on MH blood agar. Infected tissues were washed in sterile phosphate buffered saline (PBS), cut into 1 inch sections and homogenised in sterile PBS in a stomacher. Serial dilutions of the homogenised tissue were surface plated onto MH blood agar for viable counting.

All animals were handled in strict accordance with good animal practice as defined by the relevant international (Directive of the European Parliament and of the Council on the protection of animals used for scientific purposes, Brussels 543/5) and local (Moredun Research Institute, Edinburgh, UK) animal welfare guidelines. All animal work was approved by the local ethical review committee of the Moredun Research Institute, Edinburgh, UK (Permit E37/14, Project licence number 60/4471) and was licensed by the UK Government Home Office under the Animals (Scientific Procedures) Act 1986.

### Microbiology and histopathology

Ileal and colonic tissue was fixed in 10% buffered formalin solution and processed by routine methods prior to embedding in paraffin wax. Subsequently the tissue was sectioned at 5 μm and stained with haematoxylin and eosin for assessment by light microscopy.

Immunohistochemical localisation of *Campylobacter* spp. bacteria was performed on serial sections of ileal and colonic samples which were mounted onto Superfrost™ slides (Menzel-Gläser) and allowed to dry before placing in a 37 °C oven overnight. Sections were dewaxed in xylene and rehydrated through graded alcohols to 95% alcohol, prior to blocking endogenous tissue peroxidase activity with 3% hydrogen peroxide in methanol (v/v) for 20 min at room temperature. Subsequent to this, slides were washed in water prior to antigen retrieval which was performed by placing the slides in an autoclave for 10 min in citrate buffer pH 6.0 at 121 °C. Slides were left to cool to 50 °C and were washed twice in PBS containing 0.05% Tween-20 (PBST) before blocking non-specific binding with 25% normal goat serum in PBST for 30 min at room temperature. Slides were incubated overnight at 4 °C with mouse monoclonal BGN/2E10 (Abcam, UK) directed against *C. jejuni* at 1.7 μg/ml. An identical mouse isotype (IgM) antibody (Sigma M5909) was used at equivalent dilution as a negative control. Slides were washed in PBST twice before visualisation of primary antibody by applying Envision polymer (goat α-mouse-HRP) for 30 min, washed twice with PBST prior to adding Vector Nova red (Vector labs) for 10 min and subsequently washed with water. Slides were counterstained with haematoxylin for 1 min, washed in water, dehydrated through alcohols then rinsed in xylene before mounting sections in DPX (Sigma). All samples were examined by light microscopy.

### Analysis of tissue integrity

Intestinal tissue mounted vertically in Ringers solution was held in a secure chamber. The muscle layer was dissected off from the mucosa before mounting, this was to ensure that only the intestinal mucosal tissue ion movement was measured. The exposed area of tissue was 0.385 cm^2^ and the solutions were circulated using a gas lift with carbogen. Sylgard rings were used to minimise edge damage. Short circuit current (I_SC_) was monitored whilst voltage-clamping (VCC600 amplifier, Physiologic Instruments) the epithelium at 0 mV, with the mucosal bath as ground. The tissue was perfused by a mixture of 5% CO_2_ and 95% O_2_ for the duration of the experiment and the electric current measured using electrodes. Any change in the I_SC_ was detected as a change in baseline current on a graphical interface. Trans-epithelial resistance (R_T_) was calculated using Ohm’s law from voltage pulses of 1 mV for 0.35 msec. All readings were automatically corrected for electrode offsets and solution resistance with a Powerlab/8SP (AD Instruments). The bathing solutions (pH 7.4) were gassed at 37 °C and contained 113 mM NaCl, 4.5 mM KCl, 25 mM NaHCO_3_, 1.2 mM Na_2_HPO_4_, 1.1 mM CaCl_2_, 1.2 mM MgCl_2_, 10 mM glucose. Reduced chloride Ringers (18 mM chloride) were the same as the standard Ringers except the NaCl was replaced with equimolar sodium gluconate and the CaCl_2_ was increased to 5.7 mM (to compensate for the chelating effect of gluconate on calcium). Chloride conductance at apical membranes was measured using a chloride gradient with reduced chloride in the mucosal bath.

### Analysis of *C. jejuni* infection dynamics using wild-type isogenic tagged strains (WITS)

*C. jejuni* WITS strains CC001 and CC003 are derivatives of M1 that are phenotypically identical but can be distinguished by the presence of 40 bp DNA tags inserted into a pseudogene^25^. The abundance of each strain present in a mixed population can be determined by qPCR as described previously^25^. Briefly, total DNA was extracted and a qPCR was performed using primers CC069 and either CC065 (strain CC001) or CC067 (strain CC003) (Table S3). For each primer pair a standard curve of log_10_ (genome copies) *vs* C_T_ (threshold cycle) was generated by linear regression using known quantities of DNA prepared from each strain (~10^1^ to 10^7^ copies). The regression lines were used to calculate the competitive index (CI) score.

### Tn mutant library screen during piglet infection

A *mariner* Tn mutant library suitable for read-out by Tn insertion site sequencing (Tn-seq) was generated in *C. jejuni* M1cam^26^ (derivative of M1 as reported in by Friis *et al*.^44^). Briefly, the *Himar1*-C9 transposase was purified as described in ref. 45 and used for the mutagenesis of *C. jejuni* M1cam genomic DNA with the pSV006 plasmid (*mariner* Tn element carrying a chloramphenicol resistance cassette with *Mme*I restriction sites in the inverted repeats). The mutagenized DNA was repaired by T4 DNA polymerase and *E. coli* DNA ligase and introduced into *C. jejuni* M1cam through an adapted biphasic natural transformation method^46^,^47^. Tn mutants that were obtained by selective plating were pooled to generate a large Tn mutant library of 9,951 unique Tn insertion mutants^22^.

The *C. jejuni* M1cam Tn mutant library was plated onto MH blood agar plates, grown for 48 h, re-plated onto fresh MH blood agar, grown for another 48 h, and harvested in PBS. Two groups of piglets (isolator 1: *n* = 11 and isolator 2: *n* = 10) were infected with 1 ml (=4.7 × 10^8^ CFU) of the Tn mutant library suspension per piglet. Five days p.i., piglets were euthanized and faeces were collected from the last inch of the rectum, mixed with 5–10 ml of PBS, and 100 μl of this suspension was plated on MH blood agar. After 3 days, the recovered Tn mutants were harvested from plates in PBS and DNA was extracted using Genomic-tip 20/G columns (Qiagen).

Tn-seq^27^ was used to determine the relative abundance of each Tn mutant within the *C. jejuni* M1cam Tn library, essentially as described in ref. 48 with minor adjustments. Briefly, Tn mutant library genomic DNA was fragmented by *Mme*I restriction digestion, custom Tn-seq adapters with inline barcodes were ligated to the *Mme*I fragments, and Tn flanking sequences were amplified by PCR (see Table S3 for an overview of the oligonucleotides used). PCR amplification was performed using NEBNext High-Fidelity 2X PCR master mix (New England Biolabs). Tn-seq sequencing libraries were sequenced (50 bp single-end) on the Illumina HiSeq 2500 platform at Cancer Research UK (Cambridge). FastQ files were demultiplexed using the FastX toolkit barcode splitter and further analysed using ESSENTIALS^49^. The following analysis parametere were used in the ESSENTIALS analysis: sequence reads were aligned with a minimal match of 16 nt to the *C. jejuni* M1cam reference genome^26^, repeat regions were filtered, reads mapping to the 3′ end of the gene were removed, genomic position bias was corrected through Loess normalisation, read counts were normalised with the trimmed mean of M-values normalisation method (TMM). In the implemented EdgeR statistical analysis part of ESSENTIALS, the dispersion was estimated with the quantile-adjusted conditional maximum likelihood method and the variance was modelled using moderate tagwise dispersion. To identify genes of which Tn mutants showed attenuated or enriched infection of piglets, read counts were collected per gene and compared between output (piglets group 1; *n* = 4 and piglets group 2; *n* = 5) and the input (inoculum; *n* = 3). Selection criteria were as follows: genes represented >100 sequence reads in the input, a log_2_ fold-change (FC) of <−2 (attenuated survival) or >3 (enhanced survival), Benjamini & Hochberg false discovery rate <0.05, and 2 or more Tn mutants showing a log_2_ fold-change of <−2 or >3 (analysed using a custom Python script). The log_2_ fold-change cut-off was selected based on an MA-plot (Fig. S4).

In addition, 512 genes that were required for *in vitro* growth (“fitness” genes) were eliminated from our analysis; identified by analysing 23,334 unique *C. jejuni* M1 Tn insertion mutants^22^ using ESSENTIALS^49^. PCA of reads per Tn mutant, and hierarchical clustering (single linkage Euclidean distance) of log_2_ FC output/input was performed in the CLC Main Workbench v7.6.4. Functional class enrichment of genes implicated during infection of piglets was analysed using a Fisher-exact test and corrected for multiple testing using Q-value^50^. Visualization of genes required for colonisation or infection during piglet infections was performed in DNAplotter^51^.

### Sequencing data

Tn-seq sequencing data has been deposited in the European Nucleotide Archive (http://www.ebi.ac.uk/ena) and are available *via* study accession number PRJEB14716.

### Statistical analysis

Statistical analysis was performed in GraphPad Prism v6.

## Additional Information

**How to cite this article:** Vries, S. P. W.d. *et al*. Analysis of *Campylobacter jejuni* infection in the gnotobiotic piglet and genome-wide identification of bacterial factors required for infection. *Sci. Rep.* **7**, 44283; doi: 10.1038/srep44283 (2017).

**Publisher's note:** Springer Nature remains neutral with regard to jurisdictional claims in published maps and institutional affiliations.

## Supplementary Material

Supplementary Information

Supplementary Table S1
