# Supplementary material for: Analysis of Campylobacter jejuni infection in the gnotobiotic piglet and genome-wide identification of bacterial factors required for infection
Source: Sci Rep. 2017 Mar 10;7:44283. doi: 10.1038/srep44283 (PMC5345035; doi:10.1038/srep44283)
Supplement: Supplementary Information [file srep44283-s1.pdf]

Supplemental information

Analysis of *Campylobacter jejuni* infection in the gnotobiotic piglet and genome-wide identification of bacterial factors required for infection

Stefan P.W. de Vries[1#], Aileen Linn[2#], Kareen Macleod[2#], Amanda MacCallum[2], Simon P. Hardy[3], Gill Douce[4], Eleanor Watson[5], Mark P. Dagleish[5], Hal Thompson[2], Andy Stevenson[2], David Kennedy[5], Abiyad Baig[1a], Chris Coward[1b], Duncan J. Maskell[1], David G. E. Smith[6&], Andrew J. Grant[1&*], Paul Everest[2&*].

[1]Department of Veterinary Medicine, University of Cambridge, Madingley Road, Cambridge, United Kingdom

[2]University of Glasgow, Veterinary School, Glasgow, United Kingdom

[3]School of Pharmacy and Biomolecular Sciences, University of Brighton, United Kingdom

[4]Institute of Infection, Immunity and Inflammation, University of Glasgow, United Kingdom

[5]Moredun Research Institute, Edinburgh, United Kingdom

[6]Heriot-Watt University, School of Life Sciences, Edinburgh, Scotland, United Kingdom

[a]Current address: School of Veterinary Medicine and Science, University of Nottingham, Sutton Bonnington, Leicestershire, United Kingdom

[b]Current address: Discuva Ltd, The Merrifield Centre, Rosemary Lane, Cambridge, United Kingdom

[*]To whom correspondence should be addressed. E-mail: Paul.Everest@glasgow.ac.uk or ajg60@cam.ac.uk

[#]Joint first authors

[&]Joint senior authors

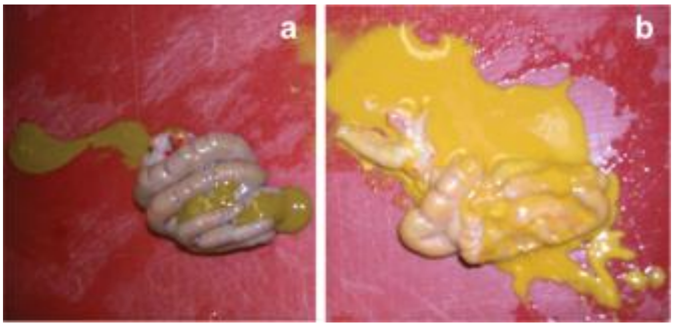

Figure S1. ***C. jejuni*** **infection induces watery diarrhoea.** Organs were obtained 4 days post-infection (p.i.) with *C. jejuni* strain 81-176. (**a**) Colonic tissue with watery diarrhoea. (**b**) Ileal tissue with watery diarrhoea.

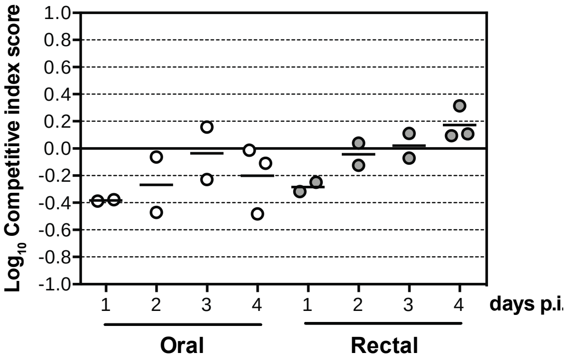

Figure S2. Stable recovery of *C. jejuni* M1 wild type isogenic tagged strains (WITS) in infected gnotobiotic piglets. After oral or rectal infection with two *C. jejuni* M1 WITS (mixed in a 1:1 ratio), faeces were collected from two or more piglets at 1, 2, 3, and 4 days p.i. The proportion of each WITS in the input and samples was determined by qPCR and used to calculate the competitive index (CI) of the two strains. Any bias towards one WITS or the other was less than half a log.

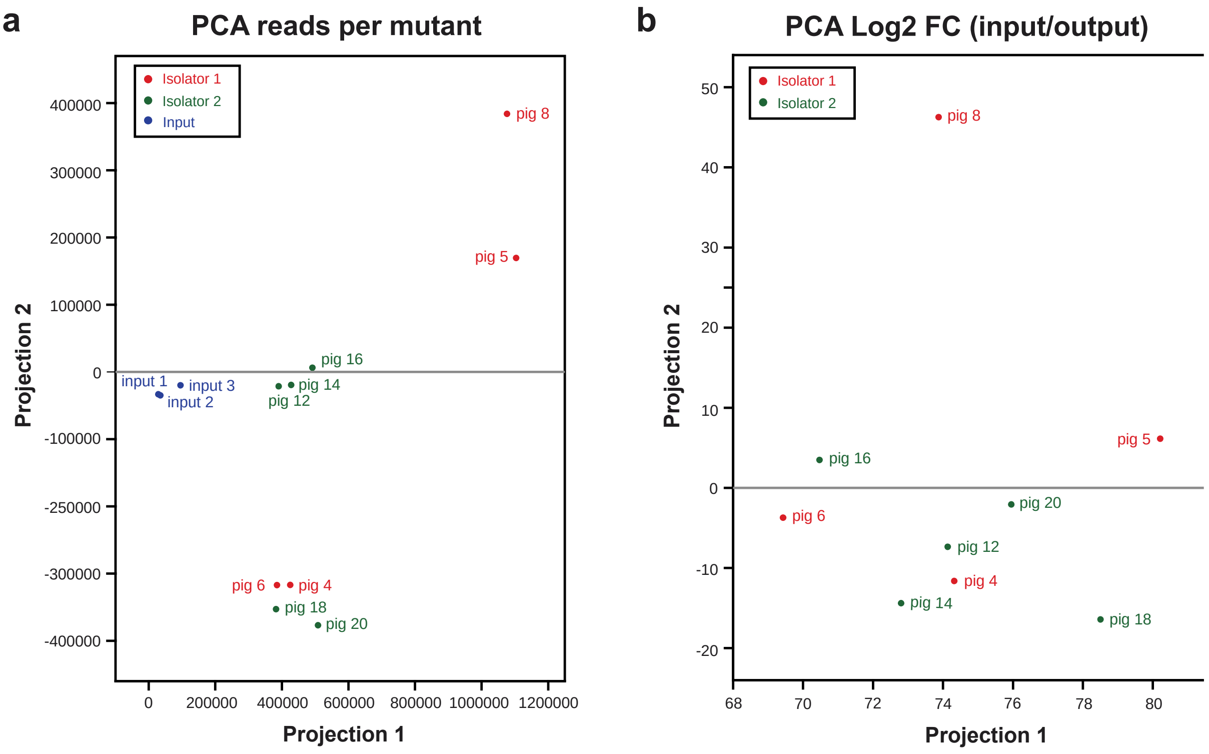

Figure S3. Characterisation of Tn mutant library behaviour in piglets housed in two separate isolators. (**a**) Principal component analysis (PCA) of read counts per individual Tn mutant showed clustering of input samples whereas no isolator-specific grouping was observed. (**b**) PCA analysis based on $\log_2$ fold-change reads per gene (output/input) did not show isolator-specific clustering of piglets. Fold-change was calculated per piglet relative to the average read count of the input samples. Piglet 8 was found to be responsible for the largest variance within the sample set, this was due to a lower number of Tn mutants being recovered from this piglet compared to others.

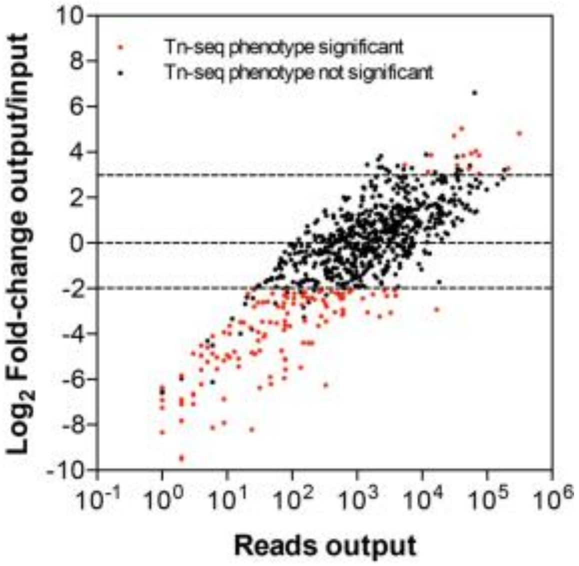

Figure S4. Log$_2$ fold-change between reads output and input (average) per gene.

Genes that fulfilled the selection criteria: > 100 sequence reads in the input, a log$_2$ fold-change of <-2 (attenuated survival) or >3 (enhanced survival), Benjamini & Hochberg false discovery rate < 0.05, and 2 or more Tn mutants showing a Log$_2$ fold-change of < -2 or > 3.

Table S2. Bacterial strains and plasmids used in this study.

Strain or plasmid	Relevant genotype or description	Source or reference
C. jejuni 11168 (original)	Original minimally passaged human enteritis isolate	Gift from Prof. Diane Newell
C. jejuni 81-176	Human campylobacteriosis outbreak isolate	[1]
C. jejuni L115	Isolate from paediatric diarrhea case	[2]
C. jejuni M1 CC001	Wild-type isogenic-tagged strain (WITS) labeled with tag 1	[3]
C. jejuni M1 CC003	Wild-type isogenic-tagged strain (WITS) labeled with tag 2	[3]
C. jejuni M1cam	Derivative of original M1 isolate; isolated from diarrheic stools of a researcher that became infected after visiting a poultry processing plant	[4, 5]
C. jejuni M1cam Tn library	Tn mutant library consisting of 9,951 unique Tn insertion mutants	[6]
pSV006	*Mariner* Tn (chloramphenicol resistance) donor plasmid; pJET1.2 backbone (Fermentas)	[6]

References

1. Korlath JA, Osterholm MT, Judy LA, Forfang JC, Robinson RA (1985) A point-source outbreak of campylobacteriosis associated with consumption of raw-milk. J Infect Dis 152: 592-596.
2. Everest P, Goossens H, Butzler J-P, Lloyd D, Knutton S, Ketley J, Williams PH (1992) Differentiated Caco-2 cells as a model for enteric invasion by *Campylobacter jejuni* and *Campylobacter coli*. J Med Microbiol 37: 319-325.
3. Coward C, van Diemen PM, Conlan AJ, Gog JR, Stevens MP, Jones MA, Maskell DJ (2008) Competing isogenic *Campylobacter* strains exhibit variable population structures in vivo. Appl Environ Microbiol 74: 3857-3867.
4. de Vries SP, Gupta S, Baig A, L'Heureux J, Pont E, Wolanska DP, Maskell DJ Grant AJ (2015) Motility defects in *Campylobacter jejuni* defined gene deletion mutants caused by second-site mutations. Microbiology 161: 2316-2327.
5. Friis, C, Wassenaar, TM, Javed, MA, Snipen, L, Lagesen, K, Hallin, PF, Newell, DG, Toszeghy, M, Ridley, A. & other authors (2010). Genomic characterization of *Campylobacter jejuni* strain M1. PLoS One 5, e12253.

6. de Vries, SP. *et al.* Genome-wide fitness analyses of the foodborne pathogen *Campylobacter jejuni* in *in vitro* and *in vivo* models. Preprint available at *BioRxiv* http://dx.doi.org/10.1101/085720 (2016).

Table S3. Oligonucleotides used in this study.

Primer name	Description	Sequence 5' to 3' ([a])
CC065	WITS tag 1 forward primer	tgggctggtgagcgttat
CC067	WITS tag 2 forward primer	cgttgggtgtgtagctggt
CC069	WITS tag 1-2 reverse primer	atatgtgcagggcgtattgc
PBGSF23	Tn-seq amplification	CAAGCAGAAGACGGCATACGAAGACCGGGGACTTATCATCCAACCTGT
PBGSF31	Tn-seq amplification	AATGATACGGCGACCACCGAGATCTACACTCTTTCCCTACACGACGCTCTTCCGATCT
PBGSF29	Tn-seq adapter B	TTCCCTACACGACGCTCTTCCGATCTCGATGTNN
PBGSF30	Tn-seq adaptor B	P-ACATCGAGATCGGAAGAGCGTCGTGTAGGGAAAGAGT-P
PBGSF29	Tn-seq adapter D	TTCCCTACACGACGCTCTTCCGATCTTGACCANN
PBGSF30	Tn-seq adaptor D	P-TGGTCAAGATCGGAAGAGCGTCGTGTAGGGAAAGAGT-P
PBGSF29	Tn-seq adapter E	TTCCCTACACGACGCTCTTCCGATCTAGTCAANN
PBGSF30	Tn-seq adaptor E	P-TTGACTAGATCGGAAGAGCGTCGTGTAGGGAAAGAGT-P
PBGSF29	Tn-seq adapter J	TTCCCTACACGACGCTCTTCCGATCTTAGCTTNN
PBGSF30	Tn-seq adaptor J	P-AAGCTAAGATCGGAAGAGCGTCGTGTAGGGAAAGAGT-P
PBGSF29	Tn-seq adapter K	TTCCCTACACGACGCTCTTCCGATCTGGCTACNN
PBGSF30	Tn-seq adaptor K	P-GTAGCCAGATCGGAAGAGCGTCGTGTAGGGAAAGAGT-P
PBGSF29	Tn-seq adapter L	TTCCCTACACGACGCTCTTCCGATCTCTTGTANN
PBGSF30	Tn-seq adaptor L	P-TACAAGAGATCGGAAGAGCGTCGTGTAGGGAAAGAGT-P
PBGSF29	Tn-seq adapter M	TTCCCTACACGACGCTCTTCCGATCTAGTTCCNN
PBGSF30	Tn-seq adaptor M	P-GGAACTAGATCGGAAGAGCGTCGTGTAGGGAAAGAGT-P
PBGSF29	Tn-seq adapter O	TTCCCTACACGACGCTCTTCCGATCTCCGTCCNN
PBGSF30	Tn-seq adaptor O	P-GGACGGAGATCGGAAGAGCGTCGTGTAGGGAAAGAGT-P
PBGSF29	Tn-seq adapter Q	TTCCCTACACGACGCTCTTCCGATCTGTGAAANN
PBGSF30	Tn-seq adaptor Q	P-TTTCACAGATCGGAAGAGCGTCGTGTAGGGAAAGAGT-P
PBGSF29	Tn-seq adapter R	TTCCCTACACGACGCTCTTCCGATCTGTGGCCNN
PBGSF30	Tn-seq adaptor R	P-GGCCACAGATCGGAAGAGCGTCGTGTAGGGAAAGAGT-P
PBGSF29	Tn-seq adapter S	TTCCCTACACGACGCTCTTCCGATCTGTTTCGNN
PBGSF30	Tn-seq adaptor S	P-CGAAACAGATCGGAAGAGCGTCGTGTAGGGAAAGAGT-P
PBGSF29	Tn-seq adapter T	TTCCCTACACGACGCTCTTCCGATCTCGTACGNN

[a] Custom Tn-seq adapters are generated by annealing PBGSF29 and PBGSF30 oligonucleotides; inline barcode sequences are underlined.
